# Profiles of Essential Oils and Correlations with Phenolic Acids and Primary Metabolites in Flower Buds of *Magnolia heptapeta* and *Magnolia denudata* var. *purpurascens*

**DOI:** 10.3390/molecules27010221

**Published:** 2021-12-30

**Authors:** Hyejin Hyeon, Ho Bong Hyun, Boram Go, Sung Chun Kim, Yong-Hwan Jung, Young-Min Ham

**Affiliations:** Biodiversity Research Institute, Jeju Technopark, Seogwipo, Jeju 63608, Korea; hhj2065@jejutp.or.kr (H.H.); hyebong@jejutp.or.kr (H.B.H.); boram01@jejutp.or.kr (B.G.); sckim@jejutp.or.kr (S.C.K.); yhjung@jejutp.or.kr (Y.-H.J.)

**Keywords:** *Magnolia heptapeta*, *Magnolia denudata* var. *purpurascens*, flower bud, essential oil, phenolic acid, primary metabolite, metabolomics, multivariate analysis

## Abstract

*Magnolia* flower buds are a source of herbal medicines with various active compounds. In this study, differences in the distribution and abundance of major essential oils, phenolic acids, and primary metabolites between white flower buds of *Magnolia heptapeta* and violet flower buds of *Magnolia denudata* var. *purpurascens* were characterised. A multivariate analysis revealed clear separation between the white and violet flower buds with respect to primary and secondary metabolites closely related to metabolic systems. White flower buds contained large amounts of monoterpene hydrocarbons (MH), phenolic acids, aromatic amino acids, and monosaccharides, related to the production of isoprenes, as MH precursors, and the activity of MH synthase. However, concentrations of β-myrcene, a major MH compound, were higher in violet flower buds than in white flower buds, possibly due to higher threonine levels and low acidic conditions induced by comparatively low levels of some organic acids. Moreover, levels of stress-related metabolites, such as oxygenated monoterpenes, proline, and glutamic acid, were higher in violet flower buds than in white flower buds. Our results support the feasibility of metabolic profiling for the identification of phytochemical differences and improve our understanding of the correlated biological pathways for primary and secondary metabolites.

## 1. Introduction

*Magnolia* is the largest genus in the family Magnoliaceae, with 210 flowering plants distributed in East and Southeast Asia [1]. Among many members of the genus, *Magnolia heptapeta* (Buc’hoz) Dandy and *Magnolia denudata* var. *purpurascens* (Maxim.) Rehder & E.H. Wilson are widely used as ornamental trees in South Korea; these species are characterised by highly fragrant and large cup-shaped flowers [2]. Furthermore, these two *Magnolia* species are regarded as medicinal plants owing to their multiple biological effects, including antioxidant, antidermatophytic, and antimicrobial activities [3,4]. *M. heptapeta* is utilised commercially due to its pharmacological safety, and *M. denudata* var. *purpurascens* is a natural source of medicinal products with no side effects [4,5].

The dried flower buds of *Magnolia*, commonly called Flos Magnoliae (also called Shin-I or Xin-yi), are a traditional herbal medicine used for the treatment of headaches, nasal congestion, gastrointestinal disorders, anxiety, and allergic rhinitis [6,7]. Previous studies have shown that various biological activities of *Magnolia* flower buds are related to their secondary metabolites, including essential oils and phenolic acids [7,8,9]. Although *Magnolia* essential oil possesses only a small fraction of the total secondary metabolites in the species, it is regarded as one of the major biologically active substances [7,10,11]. Phenolic acids are natural antioxidants in many herbal plants [12]. In addition, these secondary metabolites are commonly produced by central primary metabolic pathways, such as glycolysis, tricarboxylic acid (TCA) cycle, and amino acid and phenylpropanoid pathways. Thus, capturing the relevant primary and secondary metabolic profiles is important for understanding variation and divergence in the relative compositions of medicinal plants and for enhancing organoleptic and nutritional traits [13].

Metabolic profiling is widely employed to provide a holistic view of the chemical constituents of various medicinal plants [14,15,16]. Combined with chemometric and statistical techniques, such as principal component analysis (PCA), partial least-squares discriminant analysis (PLS-DA), and correlation analyses, metabolomics provides a comprehensive overview of metabolic data and biological processes [17,18]. These approaches are useful for obtaining information about relationships between primary and secondary metabolism by the detection of differences in phytochemical contents related to metabolic networks [18].

The relationship between phenolics and primary metabolites in white and violet *Magnolia* flowers has been investigated by metabolic profiling [19]. However, to the best of our knowledge, metabolic profiling data for phenolic acids and primary metabolites in combination with essential oils in *M. heptapeta* and *M. denudata* var. *purpurascens* flower buds have not been reported. Hence, the objective of this study was to evaluate the interactions between major secondary metabolites (essential oils and phenolic acids) and low-molecular-weight primary metabolites (amino acids, organic acids, sugars, sugar alcohols, and an inorganic acid) in *M. heptapeta* and *M. denudate* var. *purpurascens* flower buds. In this study, essential oils and low-molecular-weight primary metabolites were analysed by gas chromatography-mass spectrometry (GC-MS), and phenolic acids were quantified by high-performance liquid chromatography (HPLC). Furthermore, metabolic differences were assessed by multivariate analyses, including PCA and PLS-DA. A correlation analysis was performed to evaluate the relationships among metabolites, and metabolic mapping was conducted to investigate the differences in metabolites related to metabolic pathway. The profiles of essential oils, phenolic acids, and primary metabolites in *M. heptapeta* and *M. denudata* var. *purpurascens* flower buds may help in understanding the relationship among these metabolites.

## 2. Results and Discussion

### 2.1. Composition and Contents of Essential Oils

The capacity to produce essential oils varies greatly among medicinal plant species [20]. Moreover, the constituents of volatile oils differ among *Magnolia* genotypes [9]. In this study, the composition and contents of essential oils were compared in two types of *Magnolia* flower buds by GC-MS (Figure S1). The essential oil yields of white and violet flower buds were 0.35% and 0.2% (*w*/*w*), respectively. These results revealed that the hydro-distillation of white and violet flower buds generated different essential oil yields.

GC-MS data indicated that 30 compounds accounted for 93.24% and 87.51% of compounds in white and violet flower buds, respectively. The area percentages and retention indices (RI) of the identified compounds are presented in Table 1. The dominant constituents of the essential oils were monoterpene hydrocarbons (MHs), accounting for 58% of compounds in white flower buds and 54% in violet flower buds. The most notable difference was the percentage of β-myrcene, which was 1.73-times higher in violet flower buds (17.19%) than in white flower buds (9.94%). This compound has many biological activities, including antioxidant, anti-inflammatory, anticancer, analgesic, and anti-aging effects [21]. However, the concentrations of α-pinene and β-pinene were 1.31- and 1.68-times higher in white flower buds (6.33% and 12.92%) than in violet flower buds (4.83% and 7.67%, respectively). The sabinene content was slightly higher in white flower buds (15.19%) than in violet flower buds (14.98%). The detection of α-pinene, β-pinene, and sabinene in *Magnolia* flower buds, which have important physiological effects and practical applications, is consistent with previous findings. Research on essential oils of *Magnolia acuminate* indicated that α-pinene and β-pinene were the main constituents [22]. Moreover, in essential oil of *Magnolia biondii*, the major chemical components were α-pinene, β-pinene, and sabinene [23]. Regarding these compounds, another study found that α-pinene and β-pinene contents have significantly positive correlations with those of sabinene [24].

Oxygenated monoterpenes (MO), sesquiterpene hydrocarbons (SH), and oxygenated sesquiterpenes (SO) were present at low percentages in *Magnolia* essential oils. MO contents in white and violet flower buds were 10.64% and 14.82%, respectively (Table 1). Among MO, 1,8-cineole was dominant, with percentages of 7.59% in white flower buds and 11.61% in violet flower buds. This is consistent with previous reports revealing that the major MO compound in *Magnolia* flower buds is 1,8-cineole, which has antimicrobial and anti-inflammatory properties [6,25]. SH was also evaluated as a constituent of the essential oils in white and violet flower buds at 14.53% and 14.51%, respectively. The primary SH in *Magnolia* flower buds is germacrene D, which exhibits antimicrobial activity [26]. However, there was no significant difference in the content of germacrene D between violet flower buds (7.99%) and white flower buds (7.73%). SO contents in white and violet flower buds were 10.14% and 4.54%, respectively. α-Eudesmol and β-eudesmol, two major SO compounds, showed higher values in white flower buds (2.04% and 3.66%, respectively) than in violet flower buds (0.43% and 0.91%, respectively). Previous studies have demonstrated that β-eudesmol has diverse pharmacological activities, such as anticancer and anti-inflammatory effects, as well as effects on the nervous system [27,28].

### 2.2. Quantification of Phenolic Acids by HPLC

Together with essential oil compounds, phenolic acids are regarded as efficient natural components in medicinal plants with protective effects against oxidative stress [29]. In addition, the dynamic accumulation of essential oils is correlated with the biosynthesis of phenolic acids during plant growth [30,31]. Thus, phenolic acids isolated from white and violet-coloured *Magnolia* flower buds were analysed by HPLC (Figure S2 and Table 2). Six phenolic acid compounds (chlorogenic acid, caffeic acid, coumaric acid, rutin, ferulic acid, and cinnamic acid) were present in white and violet *Magnolia* flower buds. The dominant phenolic acid identified in the *Magnolia* flower buds was rutin, which has been used as an active component in various herbal medicines with various physiological functions, such as antioxidant, antibacterial, anti-inflammatory, antiallergic, and antiviral activities [32]. In this study, the level of rutin was 1.60-times higher in white flower buds (69.14 ± 4.63 mg/g dry weight (wt.)) than in violet flower buds (43.20 ± 2.76 mg/g (wt.)). Park et al. previously reported that rutin is the predominant phenolic compound in *Magnolia* flowers, but violet flowers contain higher amounts of rutin than those in white flowers [19]. These differences in rutin contents among studies are not surprising because phytochemical contents may be affected by various factors, including genetic variation, growth stage, climate, and location [33].

The second most abundant phenolic acid in *Magnolia* flower buds was chlorogenic acid, which is found in various medicinal plants and has beneficial properties, such as antioxidant, hypoglycaemic, antiviral, and hepatoprotective activities [34]. We detected higher levels of chlorogenic acid in violet flower buds (4.37 ± 0.08 mg/g (wt.)) than in white flower buds (3.55 ± 0.29 mg/g (wt.)). This result is consistent with that of Park et al. [19]. The caffeic acid and ferulic acid concentrations were also higher in violet flower buds than in white flower buds; however, the amounts in both forms of flower bud were remarkably low. Concentrations of other minor compounds, i.e., coumaric acid and cinnamic acid, were higher in white flower buds than in violet flower buds.

### 2.3. Metabolic Profiling of Magnolia Flower Buds by PCA

Low-molecular-weight primary metabolites, such as amino acids, organic acids, sugars, and sugar alcohols, generally assemble the building blocks of secondary metabolites and are closely linked to plant metabolism [35]. However, it is not clear how these primary metabolites are linked to functional secondary metabolites (essential oils and phenolic acids) in *Magnolia* flower buds. For the comprehensive profiling of primary and secondary metabolites in *Magnolia* flower buds, 18 amino acids, 7 organic acids, 6 sugars, 2 sugar alcohols, 1 phenolic acid, and 1 inorganic acid were evaluated by GC-MS (Figure S3 and Table S1). Thus, data for 34 primary metabolites and 37 secondary metabolites were collected from white and violet flower buds.

**Figure 1:**
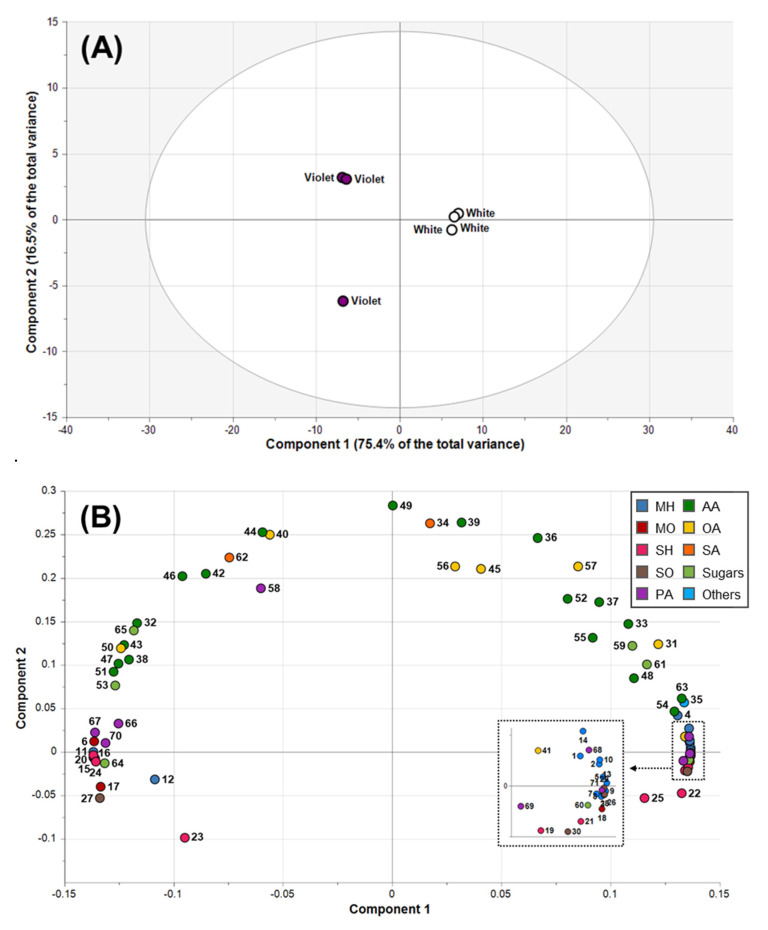
Score plots (**A**) and loading plots (**B**) of principal components 1 and 2 for the principal component analysis of essential oils, phenolic acids, and hydrophilic metabolites measured from *M. heptapeta* (white) *and M. denudata* var. *purpurascens* (violet) flower buds. MH, monoterpene hydrocarbons; MO, oxygenated monoterpene; SH, sesquiterpene hydrocarbons; SO, oxygenated sesquiterpene; PA, phenolic acids; AA, amino acids; OA, organic acids; SA, sugar alcohols. Plot annotations: 1, α-thujene; 2, α-pinene; 3, camphene; 4, sabinene; 5, β-pinene; 6, β-myrcene; 7, α-phellandrene; 8, δ-3-carene; 9, α-terpinene; 10, limonene; 11, 1,8-cineole; 12, (*Z*)-β-ocimene; 13, γ-terpinene; 14, α-terpinolene; 15, camphor; 16, 3-cyclohexen-1-ol; 17, 3-cyclohexene-1-methanol; 18, borneol acetate; 19, α-copaene; 20, β-elemene; 21, β-caryophyllene; 22, α-humulene; 23, germacrene D; 24, α-muurolene; 25, δ-cadinene; 26, elemol; 27, trans-nerolidol; 28, β-eudesmol; 29, α-eudesmol; 30, α-cadinol; 31, glycolic acid; 32, alanine; 33, valine; 34, glycerol; 35, phosphoric acid; 36, leucine; 37, isoleucine; 38, proline; 39, glycine; 40, succinic acid; 41, fumaric acid; 42, serine; 43, threonine; 44, β-alanine; 45, malic acid; 46, aspartic acid; 47, methionine; 48, pyroglutamic acid; 49, γ-aminobutyric acid; 50, threonic acid; 51, glutamic acid; 52, phenylalanine; 53, xylose; 54, asparagine; 55, glutamine; 56, shikimic acid; 57, citric acid; 58, quinic acid; 59, fructose; 60, galactose; 61, glucose; 62, inositol; 63, tryptophan; 64, sucrose; 65, raffinose; 66, chlorogenic acid; 67, caffeic acid; 68, coumaric acid; 69, rutin; 70, ferulic acid; 71, cinnamic acid.

PCA was applied to the entire dataset to determine the overall clustering pattern of two types of samples based on differences in primary and secondary metabolites (Figure 1). PCA, a well-known unsupervised technique, is widely used as a first step to objectively interpret and compare large-scale metabolic data [36]. The PCA score plot revealed that the first two principal components (PCs) explained 91.9% of the total variance (Figure 1A). In addition, PC1 explained 75.4% of the variance, clearly resolving samples into two groups corresponding to white and violet flower buds. To further investigate the main metabolites responsible for the separation between the two groups, PCA loadings were inspected (Figure 1B). Most MH, SO, phenolic acids, aromatic amino acids, and monosaccharides clustered on the right side of the loading plot, showing that these compounds were more abundant in white flower buds than in violet flower buds. The representative metabolites positive for PC1 were β-pinene, β-eudesmol, rutin, tryptophan, and galactose with eigenvector values of 0.1365, 0.1365, 0.1334, 0.1327, and 0.1359, respectively. MO, polysaccharides, serine-threonine metabolism-related amino acids, and proline metabolism-related amino acids were grouped on the left side of the loading plots, indicating higher amounts of these metabolites in violet flower buds. Notably, the metabolites that contributed negatively to PC1 were 1,8-cineole, sucrose, threonine, and proline with eigenvector values of −0.1362, −0.1315, −0.1276, and −0.1203, respectively. Our results agree with previous studies and corroborate findings that higher amounts of monosaccharides were found in white coloured *Magnolia* for production of phenolic acids while polysaccharides were hydrolysed into monosaccharides for use as an energy source [19,35].

Overall, the PCA results indicated that most MH, SO, phenolic acids, aromatic amino acids, and monosaccharides were more abundant in white flower buds than in violet flower buds. In contrast, the amounts of MO, polysaccharides, serine-threonine metabolism-related amino acids, and proline metabolism-related amino acids were higher in violet flower buds than in white flower buds.

### 2.4. Correlations among Essential Oils, Phenolic Acids, and Hydrophilic Compounds

**Figure 2:**
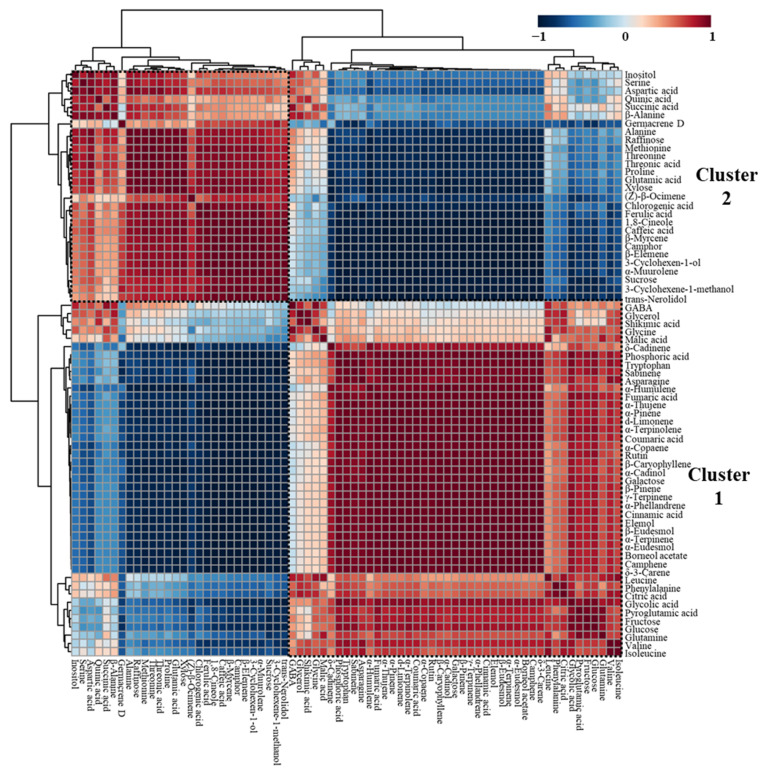
Correlation matrix of 71 metabolites from white and violet *Magnolia* flower buds. Each square indicates the Pearson’s correlation coefficient for a pair of compounds, and the correlation coefficient is represented by the shade of blue or red, as indicated on the colour scale.

Correlation analyses based on Pearson correlation coefficients provide powerful information of relationships among metabolites [35,37]. Therefore, a hierarchical cluster analysis (HCA) with Pearson correlation coefficients was performed to understand the relationships between primary and secondary metabolites in white and violet flower buds (Figure 2, Table S2). In Figure 2, the shade of blue or red represents correlation coefficients closer to −1 or 1, respectively.

Pairwise correlations between metabolites formed two major clusters (shown in dotted lines), and metabolites from the same classes, such as MH, MO, and SO, generally clustered together. Furthermore, these outcomes were consistent with the PCA results (Figure 1B). In cluster 1, most of the MH, SO, aromatic amino acids, organic acids, monosaccharides, and several phenolic acids, which had positive values on PC1 in PCA loading plots, clustered together with positive correlations. For example, β-pinene was highly correlated with β-eudesmol (*r* = 0.9984, *p* < 0.0001), tryptophan (*r* = 0.9770, *p* = 0.0008), fumaric acid (*r* = 0.9714, *p* = 0.0012), galactose (*r* = 0.9900, *p* = 0.0002), and rutin (*r* = 0.9738, *p* = 0.0010). In addition, positively correlated metabolites in cluster 2 included most of the MO, polysaccharides, serine-threonine metabolism-related amino acids, and proline metabolism-related amino acids, and those with negative values on PC1 in PCA loading plots. For instance, 1,8-cineole was highly correlated with sucrose (*r* = 0.9407, *p* = 0.0052), threonine (*r* = 0.9179, *p* = 0.0098), and proline (*r* = 0.9135, *p* = 0.0109). However, β-myrcene, the major MH in *Magnolia* flower buds, was also assigned to cluster 2, rather than cluster 1. β-Myrcene was significantly positively correlated with 1,8-cineole (*r* = 0.9970, *p* < 0.0001) and was negatively correlated with β-pinene (*r* = −0.9996, *p* < 0.0001).

Overall, the observed metabolite-to-metabolite correlations reflected significant differences in metabolites between the two types of *Magnolia* flower buds and closely related metabolic pathways.

### 2.5. Classification of Magnolia Flower Buds Based On PLS-DA

**Figure 3:**
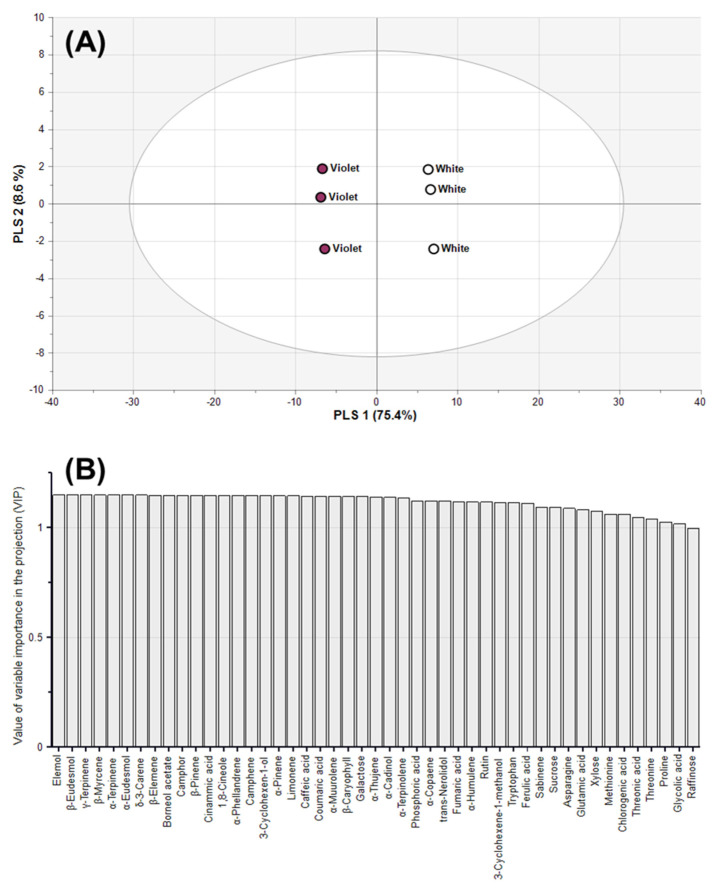
Partial least squares discriminant analysis (PLS-DA) score plots (**A**) and variable importance in projection (VIP) scores above 1.0 (**B**) for *M. heptapeta* (white) *and M. denudata* var. *purpurascens* (violet) flower buds.

Although PCA and HCA are useful tools for summarising metabolomics data, these methods have limitations in capturing the number of significantly relevant components [38,39]. Thus, we used PLS-DA to identify novel metabolites that effectively discriminated between white and violet flower buds (Figure 3). PLS-DA, a supervised technique, is suitable for maximising separation between varieties and identifying metabolite contributors for classification [38,40]. The goodness of fit in the PLS-DA model is estimated by the coefficient of determination (*R*_X_^2^), where a value closer to 1 indicates a good fit. The prediction accuracy of the PLS-DA model is explained by the cross-validation coefficient of determination (*Q*^2^), where values above 0.9 indicate an excellent model. We obtained *R*_X_^2^ and *Q*^2^ values of 0.840 and 0.994, respectively (Figure 3A). Thus, the PLS-DA clearly separated the two types of *Magnolia* flower buds with a high goodness of fit and reliable predictive ability.

**Figure 4:**
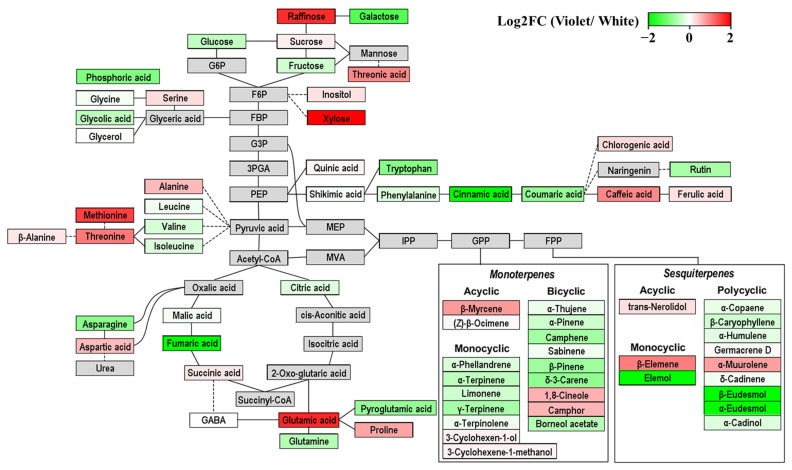
Metabolic pathway visualization and relative metabolite abundance of *Magnolia* flower buds. Fold changes (FC) from white to violet flower buds were converted into log2-transformed values (log2FC). A log2FC value range is −2 < log2FC < 2. If log2FC value is higher than zero (indicated in red), metabolite content is higher in violet flower buds than in white flower buds. If log2FC value is less than zero (indicated in green), metabolite content is higher in white flower buds than in violet flower buds.

To gain more insight into the metabolites contributing to the differences between white and violet flower buds, the variable importance in projection (VIP) scores were assessed (Figure 3B). VIP describes the residual sum of squares of the PLS weight, and values greater than 1.00 are considered highly influential. Setting VIP > 1.00 and *p*-value < 0.05 as thresholds, 47 metabolites effectively distinguished between white and violet flower buds (Figure S4). Moreover, a metabolic pathway map was produced to correlate relative metabolite contents of white and violet *Magnolia* flower buds with the biosynthetic pathway (Figure 4). The imported data was calculated by dividing the average data of violet flower buds into those of white flower buds, and then converted into log2-transformed values (log2FC). The positive and negative values of log2FC were visualised as a gradient of red and green colours, respectively. Shades of green colour represented higher amounts in white flower buds, and shades of red colour represented higher amounts in violet flower buds.

Among these influential metabolites, MH was the largest class of variables with significant discriminatory ability. Eleven MH compounds (α-thujene, α-pinene, camphene, sabinene, β-pinene, α-phellandrene, δ-3-carene, α-terpinene, limonene, γ-terpinene, and α-terpinolene) were more abundant in the white flower buds than in violet flower buds. Regarding terpene synthesis, the accumulation of carbohydrates provides carbon building blocks for isoprene production by glycolysis; these five-carbon isoprene units form the backbone structure of terpenes and are joined to C10 monoterpene and C15 sesquiterpene substrates with various synthases [41,42]. Of these enzymes, monoterpene synthase produces various cyclic and acyclic monoterpenes by multiple mechanisms involving cationic intermediates and hydride shifts; however, the enzyme activity varies substantially among different conditions [43]. In particular, aromatic amino acids and phenolic acids stabilize the synthase content and promote the rearrangement of isoprene units [44]. Interestingly, our results revealed that the levels of galactose, tryptophan, rutin, cinnamic acid, and coumaric acid were higher in white flower buds than in violet flower buds, and monosaccharides, aromatic amino acids, and phenolic acids were representative differential metabolites linked to MH synthesis. A higher concentration of galactose may be required as a carbon source to generate isoprene units. As a consecutive metabolic reaction in isoprene synthesis, MH production might be catalysed by monoterpene synthases with high levels of tryptophan, rutin, cinnamic acid, and coumaric acid in white flower buds [44].

However, we noted that only the content of β-myrcene was comparatively higher in violet flower buds. Unlike other MH shown to have higher contents in white flower buds, β-myrcene has an acyclic unsubstituted monoterpene form [45]. In addition, myrcene biosynthesis needs characteristic monoterpene synthases, and sequences of these monoterpene synthases feature higher contents of threonine and serine residues and low contents of acidic residues [43,46]. Our results also suggested that the content of threonine was elevated, whereas fumaric acid and glycolic acid, which caused acidic pH conditions, were significantly low in violet flower buds compared to white flower buds. Hence, these observations implied that higher concentration of threonine and lower concentrations of some organic acids contributed to the changes of monoterpene synthesis mechanisms, increasing β-myrcene production in violet flower buds. Furthermore, different content pattern of β-myrcene compared with other MH in *Magnolia* flower buds might be due to the specialized structure of this compound.

The MO group showed significant differences between *Magnolia* species, with higher amounts of 1,8-cineole, camphor, 3-cyclohexane-1-ol, and 3-cyclohexane-1-methanol and lower amounts of borneol acetate in violet flower buds than in white flower buds. In particular, the high abundance of 1,8-cineole, the major MO in *Magnolia* flower buds, normally reflects an oxidative stress response mechanism under a high level of reactive oxygen species (ROS) [47]. Stress conditions in plants can also result in the accumulation of proline, an oxidative stress biomarker [48]. Moreover, proline metabolism is usually derived from the synthesis of glutamic acid, which acts as a precursor of proline [49]. Our results showed that levels of stress-related amino acids, proline and glutamic acid, were higher in violet flower buds, and similar to levels of 1,8-cineole. Thus, we suggest that defensive mechanisms against ROS stimulate the production of oxidative stress-related MO compounds (1,8-cineole) and amino acids (proline and glutamic acid) in violet flower buds.

Various patterns were observed for SH levels. Specifically, β-caryophyllene, α-humulene, and α-coupaene were more abundant in white flower buds, and β-elemene and α-muurolene were more abundant in violet flower buds. This may be explained by the fact that sesquiterpenes generated by sesquiterpene synthases have more diverse structures and contents than those of monoterpenes due to the increased number of possible combinations with five carbons of isoprene units [50]. However, SO levels clearly differed between the two *Magnolia* flower buds, with significantly higher levels of β-eudesmol, α-eudesmol, α-cadinol, and elemol in white flower buds. In violet flower buds, only trans-nerolidol levels were higher than those in white flower buds. Although the significance of these metabolites is not yet apparent, these results indicated that the biosynthesis of major SO compounds was higher in white flower buds than in violet flower buds.

## 3. Materials and Methods

### 3.1. Preparation of Plant Materials

Healthy flower buds of *Magnolia heptapeta* (Buc’hoz) Dandy and *Magnolia denudata* var. *purpurascens* (Maxim.) Rehder & E.H. Wilson were collected in the Namwon region of Jeju, Korea, in February 2021. The harvested samples were mixed; one kilogram of each sample was stored at 4 °C to determine the chemical composition of the essential oils. To analyse phenolic acids and low-molecular-weight hydrophilic compounds, the remaining flower buds were dried in a drying oven at 45 °C for 72 h. The dried plant materials were ground into a fine powder using a grinding machine.

### 3.2. Essential Oil Extraction

The essential oils were isolated from 1 kg of fresh flower buds by hydrodistillation using a Clevenger-type apparatus for 8 h. The essential oils were spontaneously separated from the aqueous layer and then moved into dark vials using a pipette. The essential oil yield based on % (*v*/*w*) was calculated on a fresh weight basis. The extracted essential oils were stored at 4 °C prior to further processing [51].

### 3.3. Chemical Composition Analysis of Essential Oil

The constituents of the essential oils were characterised using a previously reported method with slight modifications [52]. The isolated essential oils were diluted 1:1000 (*v*/*v*) with hexane, followed by filtration through a 0.50 µm PTFE filter (Advantec, Tokyo, Japan). The samples were analysed on an Agilent 7890A GC (Agilent, Palo Alto, CA, USA) equipped with an Agilent 5975C MSD. An HP-5MS capillary column (30 m × 0.25 mm i.d. × 0.25 μm film thickness; 19091S-433, Agilent) was equipped into the GC, and helium gas was used as the carrier gas at a flow rate of 1.00 mL/min. The split ratio was set to 10:1, the injection volume was 1 μL, the injection temperature was 230 °C, the standard electronic impact (EI)-MS source temperature was 230 °C, and the MS quadrupole temperature was 150 °C. The spectral data were scanned over an *m/z* mass range of 50–550, and the ionisation voltage was set to 70 eV. The oven temperature was programmed with the following gradient conditions: starting at 40 °C for 2 min, followed by ramping to 250 °C at 3 °C/min, and holding at this temperature for 10 min. ChemStation (Agilent) was used to analyse the chromatograms and mass spectra. Each volatile compound was identified by comparing the retention indices to those of n-alkanes (C8-C20) reported in the literature as well as EI-MS data reported in the literature and registered by the National Institute of Standards and Technology (NIST) [53,54,55]. Peaks were assigned when the similarity was >90%. The relative percentage areas of compounds in the samples were obtained from the chromatograms by normalising the peak area.

### 3.4. Phenolic Acid Extraction and Analysis

Phenolic acids were extracted and analysed according to a previously reported method with slight modifications [56]. A total of 100 mg of the powdered sample was placed into a 2 mL tube and mixed with 1 mL of methanol and 200 µL of 0.1 M hydrochloric acid (HCl). The samples were vortexed for 1 min and sonicated for 1 h. Next, the mixture was centrifuged at 13,000 rpm for 10 min, and 800 µL of the supernatant was transferred into a new 2 mL tube. The samples were re-extracted by adding 1 mL of methanol to the remaining pellet. After centrifugation under the same conditions described above, the supernatant was collected, concentrated, and re-dissolved in 1 mL of methanol. The resulting extract was filtered using a 0.50 µm PTFE filter (Advantec) into amber glass screw thread vials (Agilent). Phenolic acids were separated using a Waters Alliance e2695 HPLC system (Waters Corp., Milford, MA, USA), equipped with a photodiode array detector and a Zorbax CB-C18 column (250 × 4.6 mm, 5 µm particle size; Agilent). As gradient elution solvents, 0.1% acetic acid in water (solvent A) and 0.1% acetic acid in acetonitrile (solvent B) were used. The gradient elution was processed as follows: 0 min, 92% A/8% B; 2 min, 90% A/10% B; 35 min, 70% A/30% B; 50 min, 10% A/90% B; 51 min, 0% A/100% B; 60 min, 0% A/100% B; 63 min, 92% A/8% B. The sample injection volume was 10 µL, and the column flow rate and temperature were set at 1 mL/min and 40 °C, respectively. Peaks were monitored at wavelengths of 200–400 nm, and the chromatogram was obtained at 280 nm. Each phenolic acid was identified by comparing the retention time and UV absorbance spectra with those of the standards. Quantification was performed by plotting the concentrations of the standards (2.5, 5, 10, and 20. 40, 60, and 80 ppm), and the calibration curves were drawn.

### 3.5. Low-Molecular-Weight Hydrophilic Compound Extraction and Analysis

The extraction and analysis of low-molecular-weight hydrophilic compounds (amino acids, organic acids, sugars, and sugar alcohols) were carried out according to a previously described method [35]. Fifteen milligrams of dried samples were added to 1 mL of methanol: chloroform: water (2.5:1:1, *v*/*v*), and 60 μL of ribitol (0.2 mg/mL in methanol) was introduced as an internal standard (IS). Thereafter, the samples were briefly vortexed and placed in a thermo shaker at 37 °C for 30 min with shaking at 1200 rpm. The tubes were centrifuged at 13,000 rpm for 3 min, and 800 μL of the supernatant was transferred into new tubes. Next, each sample was mixed with 400 μL of deionised water, followed by centrifugation at 13,000 rpm for 5 min. The upper layer of the methanol/chloroform phase was pipetted into a fresh tube and concentrated for 3 h. The residues were freeze-dried for at least 18 h at −80 °C. For the derivatisation step, the lyophilised samples were treated with 80 μL of methoxyamine hydrochloride in pyridine (2%, *w*/*v*) and incubated at 30 °C and 1200 rpm for 90 min. Next, 80 μL of *N*-methyl-*N*-(trimethylsilyl) trifluoroacetamide in pyridine was added, and the mixture was reacted at 37 °C and 1200 rpm for 30 min. Finally, the low-molecular-weight hydrophilic compounds were analysed on an Agilent 7890A GC equipped with an Agilent 5975C MSD and separated on a CP-Sil 8CB low-bleed/MS fused-silica capillary column (30 m × 0.25 mm i.d. × 0.25 μm film thickness; CP5860, Agilent). Each 1.0 µL sample was injected with a 25:1 split ratio mode. Helium gas was used as the carrier gas at a rate of 1 mL/min. The injector, MS quadrupole, and ion source temperatures were set to 230 °C, 150 °C, and 230 °C, respectively. The oven temperature was programmed as follows: 80 °C for 2 min, followed by an increase to 320 °C at a rate of 15 °C/min, and holding at this temperature for 10 min. The spectral data were scanned at *m/z* 85–600, and the ionisation voltage was set at 70 eV. Chromatographic data were processed using ChemStation (Agilent). The identities of the compounds were confirmed by comparing the NIST and in-house libraries. For relative quantification, peak areas of metabolites based on the IS were calculated based on the selected ions.

### 3.6. Statistical Analysis

The metabolite data for the *Magnolia* flower bud samples were obtained with three biological replicates and are presented as means ± standard deviation. The resultant data were normalised with unit variance scaling and then subjected to PCA and PLS-DA using soft independent modelling of class analogy (SIMCA) (version 17.0; Umetrics, Umeå, Sweden). PCA, an unsupervised pattern recognition technique, was applied to evaluate metabolite patterns. Moreover, PLS-DA, a supervised classification method, was conducted to reveal separation between experimental groups and to obtain insight into the factors contributing to the separation. Independent Student’s *t*-tests were used for comparisons of levels of metabolites between the two groups using GraphPad Prism 8 (San Diego, CA, USA), with *p* < 0.05 as the threshold for significance. Pearson’s correlation analysis and HCA were performed using MetaboAnalyst 5.0 (www.metaboanalyst.ca) (accessed on 9 October 2021) to reveal correlations among metabolites. PathVisio software (version 3.3.0, www.pathvisio.org) (accessed on 28 December 2021) was employed to visualize metabolic pathway maps with log2-transformed experimental data. The metabolic pathway was drawn based on the *Arabidopsis thaliana pathway* in WikiPathways (www.wikipathways.org) (accessed on 28 December 2021) and reference pathway in the KEGG database (www.genome.jp/kegg/pathway.html) (accessed on 27 December 2021).

## 4. Conclusions

We performed the first comprehensive metabolic profiling of phenolic acids and primary metabolites together with essential oils in the white flower buds of *M. heptapeta* and violet flower buds of *M. denudata* var. *purpurascens*. The chemical compositions of essential oils from white and violet flower buds were notably different, with higher levels of MH and SO in white flower buds and higher levels of β-myrcene and MO in violet flower buds. A multivariate analysis of data for secondary metabolites (essential oils and phenolic acids) and primary metabolites (amino acids, organic acids, sugars, sugar alcohols, and an inorganic acid) showed that metabolite differences and correlations in *Magnolia* flower buds depended on common or closely related metabolic pathways. In white flower buds, elevated levels of aromatic amino acids and phenolic acids led to optimum conditions for the synthesis of MH, and monosaccharides were used as building blocks of isoprene units, which construct MH. In violet flower buds, higher threonine and lower acidic organic acid levels were significantly correlated with elevated β-myrcene production. Associated with the stress response mechanism, MO and proline metabolism-related amino acid levels were higher in violet flower buds than in white flower buds. Thus, these results confirmed the value of metabolic profiling using chemometric tools for detecting significant correlations between essential oils, phenolic acids, and low-molecular-weight primary metabolites in white and violet flower buds. In addition, our results provide useful information about functional essential oils and phenolic acids in *Magnolia* flower buds. When *Magnolia* flower buds are used as foods or herbal medicines, our insights are expected to contribute to the selection of sources enriched for desired nutrients.

## Figures and Tables

**Table 1:** Essential oil chemical compositions of *M. heptapeta* (white) *and M. denudata* var. *purpurascens* (violet) flower buds.

NO.	Type	Compounds	RT	RI	Relative Content (%)	
					White	Violet
1	MH	α-Thujene	8.028	925	0.552 ± 0.003	0.498 ± 0.005
2	MH	α-Pinene	8.234	930	6.328 ± 0.019	4.828 ± 0.087
3	MH	Camphene	8.787	944	0.406 ± 0.009	0.248 ± 0.003
4	MH	Sabinene	9.787	971	15.789 ± 0.055	14.58 ± 0.334
5	MH	β-Pinene	9.869	973	12.921 ± 0.052	7.668 ± 0.202
6	MH	β-Myrcene	10.581	992	9.936 ± 0.052	17.194 ± 0.143
7	MH	α-Phellandrene	11.051	1003	0.444 ± 0.002	0.363 ± 0.004
8	MH	δ-3-Carene	11.292	1009	0.196 ± 0.002	0.107 ± 0.002
9	MH	α-Terpinene	11.575	1015	1.788 ± 0.011	1.254 ± 0.003
10	MH	Limonene	12.086	1027	3.811 ± 0.006	2.948 ± 0.058
11	MO	1,8-Cineole	12.192	1029	7.589 ± 0.007	11.606 ± 0.193
12	MH	(Z)-β-Ocimene	13.039	1048	0.231 ± 0.004	0.240 ± 0.005
13	MH	γ-Terpinene	13.439	1057	4.644 ± 0.014	2.881 ± 0.029
14	MH	α-Terpinolene	14.757	1087	0.889 ± 0.006	0.818 ± 0.007
15	MO	Camphor	17.286	1143	0.238 ± 0.001	0.354 ± 0.004
16	MO	3-Cyclohexen-1-ol	18.810	1147	0.876 ± 0.004	0.954 ± 0.002
17	MO	3-Cyclohexene-1-methanol	19.451	1168	1.499 ± 0.009	1.613 ± 0.023
18	MO	Borneol acetate	23.715	1286	0.440 ± 0.003	0.295 ± 0.004
19	SH	α-Copaene	27.485	1374	0.114 ± 0.001	0.100 ± 0.003
20	SH	β-Elemene	28.209	1391	0.333 ± 0.009	0.673 ± 0.008
21	SH	β-Caryophyllene	29.274	1417	2.610 ± 0.042	2.031 ± 0.038
22	SH	α-Humulene	30.668	1451	0.660 ± 0.009	0.562 ± 0.018
23	SH	Germacrene D	31.809	1479	7.729 ± 0.037	7.986 ± 0.234
24	SH	α-Muurolene	32.638	1500	0.399 ± 0.014	0.665 ± 0.020
25	SH	δ-Cadinene	33.544	1523	2.682 ± 0.049	2.495 ± 0.089
26	SO	Elemol	34.573	1550	1.042 ± 0.004	0.193 ± 0.005
27	SO	trans-Nerolidol	35.173	1566	0.491 ± 0.010	0.586 ± 0.015
28	SO	β-Eudesmol	38.302	1650	3.658 ± 0.023	0.908 ± 0.023
29	SO	α-Eudesmol	38.420	1653	2.039 ± 0.032	0.430 ± 0.032
30	SO	α-Cadinol	38.508	1655	2.905 ± 0.017	2.426 ± 0.054
Monoterpene hydrocarbons (MH)					57.936 ± 0.118	53.627 ± 0.582
Oxygenated monoterpenes (MO)					10.642 ± 0.012	14.823 ± 0.162
Sesquiterpene hydrocarbons (SH)					14.528 ± 0.122	14.513 ± 0.403
Oxygenated sesquiterpenes (SO)					10.136 ± 0.043	4.543 ± 0.111
		Total			93.24	87.51

Each value is the mean of three replications ± standard deviation.

**Table 2:** Composition and contents of phenolic acids in *M. heptapeta* (white) *and M. denudata* var. *purpurascens* (violet) flower buds.

NO.	Name (mg/g Dry Weight)	Formula	White	Violet
1	Chlorogenic acid	C_16_H_18_O_9_	3.548 ± 0.285	4.372 ± 0.075
2	Caffeic acid	C_9_H_8_O_4_	0.186 ± 0.008	0.356 ± 0.011
3	Coumaric acid	C_9_H_8_O_3_	0.117 ± 0.003	0.065 ± 0.004
4	Rutin	C_27_H_30_O_16_	69.136 ± 4.625	43.200 ± 2.763
5	Ferulic acid	C_10_H_10_O_4_	0.132 ± 0.006	0.156 ± 0.001
6	Cinnamic acid	C_9_H_8_O_2_	0.084 ± 0.003	0.018 ± 0.001

Each value is the mean of three replications ± standard deviation.

## Data Availability

Not applicable.

## Supplementary Materials

The following are available online, Figure S1: Representative GC-MS chromatogram of essential oils from *M. heptapeta* (white) and *M. denudata* var. *purpurascens* (violet) flower buds. Peak annotation: 1, α-thujene; 2, α-pinene; 3, camphene; 4, sabinene; 5, β-pinene; 6, β-myrcene; 7, α-phellandrene; 8, δ-3-carene; 9, α-terpinene; 10, limonene; 11, 1,8-cineole; 12, (Z)-β-ocimene; 13, γ-terpinene; 14, α-terpinolene; 15, camphor; 16, 3-cyclohexen-1-ol; 17, 3-cyclohexene-1-methanol; 18, borneol acetate; 19, α-copaene; 20, β-elemene; 21, β-caryophyllene; 22, α-humulene; 23, germacrene D; 24, α-muurolene; 25, δ-cadinene; 26, elemol; 27, trans-nerolidol; 28, β-eudesmol; 29, α-eudesmol; 30, α-cadinol. Figure S2: Representative HPLC chromatogram of phenolic acids from *M. heptapeta* (white) and *M. denudata* var. *purpurascens* (violet) flower buds. Peak annotation: 1, chlorogenic acid; 2, caffeic acid; 3, coumaric acid; 4, rutin; 5, ferulic acid; 6, cinnamic acid. Figure S3: Representative GC-MS chromatogram of low-molecular hydrophilic compound in *M. heptapeta* (white) and *M. denudata* var. *purpurascens* (violet) flower buds. Peak annotation: 1, glycolic acid; 2, alanine; 3, valine; 4, glycerol; 5, phosphoric acid; 6, leucine; 7, isoleucine; 8, proline; 9, glycine; 10, succinic acid; 11, fumaric acid; 12, serine; 13, threonine; 14, β-alanine; 15, malic acid; 16, aspartic acid; 17, methionine; 18, pyroglutamic acid; 19, γ-aminobutyric acid; 20, threonic acid; 21, glutamic acid; 22, phenylalanine; 23, xylose; 24, asparagine; 25, glutamine; 26, shikimic acid; 27, citric acid; 28, quinic acid; 29, fructose-1; 30, fructose-2; 31, galactose; 32, glucose-1; 33, glucose-2; 34, inositol; 35, tryptophan; 36, sucrose; 37, raffinose. Figure S4: Box plots of significantly different metabolites (*p* < 0.05) between *M. heptapeta* (white) and *M. denudata* var. *purpurascens* (violet) flower buds. Table S1: Composition and contents (ratio/g) of low-molecular-weight hydrophilic compounds in *M. heptapeta* (white) and *M. denudata* var. *purpurascens* (violet) flower buds. Table S2: Results of the Pearson correlation analysis.

## Sample Availability

Samples of the compounds are not available from the authors.
