# Supplementary material for: Profiles of Essential Oils and Correlations with Phenolic Acids and Primary Metabolites in Flower Buds of Magnolia heptapeta and Magnolia denudata var. purpurascens
Source: Molecules. 2021 Dec 30;27(1):221. doi: 10.3390/molecules27010221 (PMC8746637; doi:10.3390/molecules27010221)
Supplement: Supplementary file 1 [file molecules-27-00221-s001.zip › molecules-1517576-supplementary/Supplementary figure_magnolia.pptx]

## Slide 1
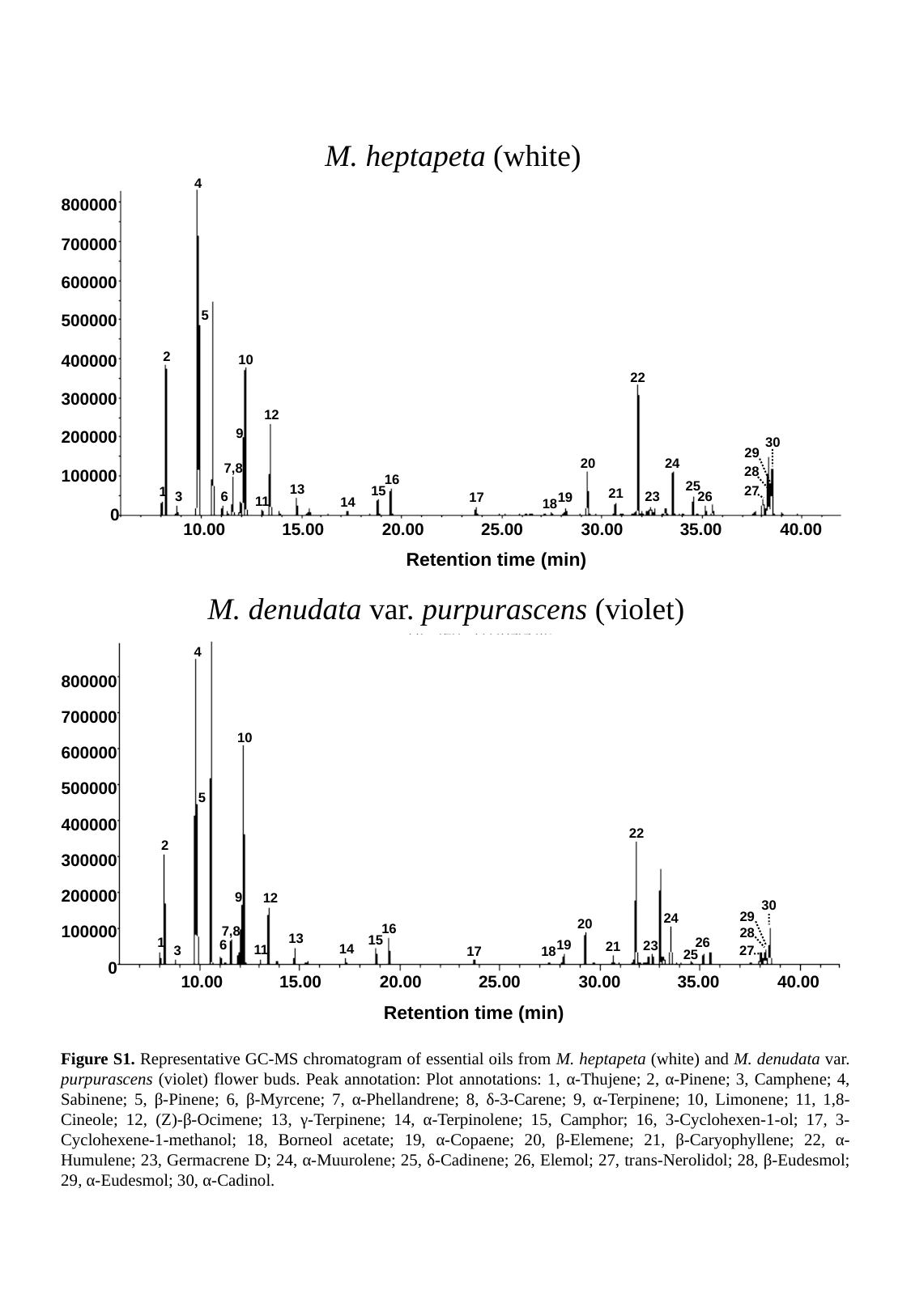

M. heptapeta (white)
4
800000
700000
600000
5
500000
2
400000
10
22
300000
12
9
200000
30
29
24
20
7,8
28
100000
16
25
13
15
27
1
21
6
3
26
23
17
19
11
14
18
0
10.00
15.00
20.00
25.00
30.00
35.00
40.00
Retention time (min)
M. denudata var. purpurascens (violet)
4
800000
700000
10
600000
500000
5
400000
22
2
300000
200000
9
12
30
29
24
20
16
100000
7,8
28
13
15
26
1
6
19
23
21
14
11
3
27
17
18
25
0
10.00
15.00
20.00
25.00
30.00
35.00
40.00
Retention time (min)
Figure S1. Representative GC-MS chromatogram of essential oils from M. heptapeta (white) and M. denudata var. purpurascens (violet) flower buds. Peak annotation: Plot annotations: 1, α-Thujene; 2, α-Pinene; 3, Camphene; 4, Sabinene; 5, β-Pinene; 6, β-Myrcene; 7, α-Phellandrene; 8, δ-3-Carene; 9, α-Terpinene; 10, Limonene; 11, 1,8-Cineole; 12, (Z)-β-Ocimene; 13, γ-Terpinene; 14, α-Terpinolene; 15, Camphor; 16, 3-Cyclohexen-1-ol; 17, 3-Cyclohexene-1-methanol; 18, Borneol acetate; 19, α-Copaene; 20, β-Elemene; 21, β-Caryophyllene; 22, α-Humulene; 23, Germacrene D; 24, α-Muurolene; 25, δ-Cadinene; 26, Elemol; 27, trans-Nerolidol; 28, β-Eudesmol; 29, α-Eudesmol; 30, α-Cadinol.

## Slide 2
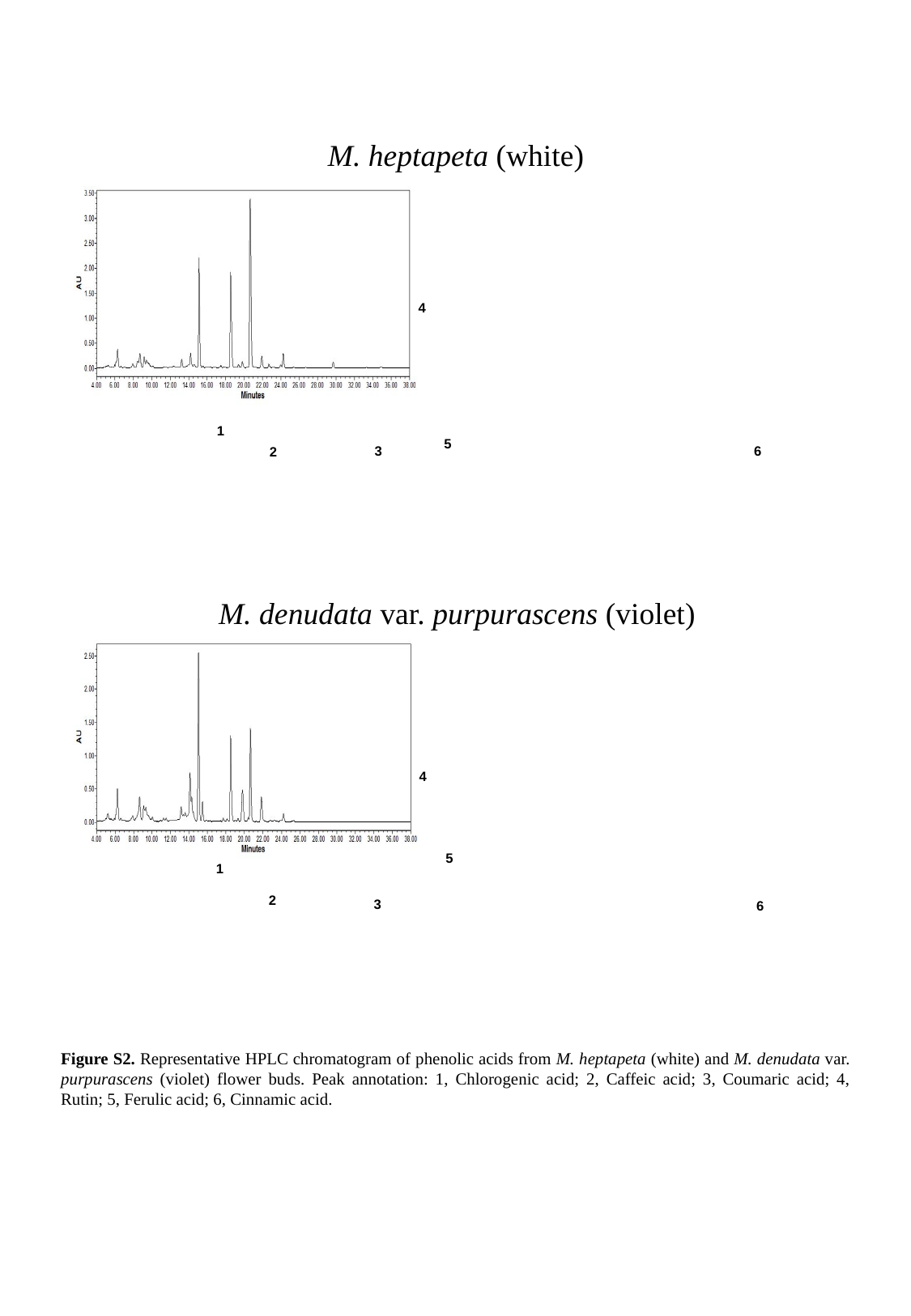

M. heptapeta (white)
4
1
5
3
6
2
M. denudata var. purpurascens (violet)
4
5
1
2
3
6
Figure S2. Representative HPLC chromatogram of phenolic acids from M. heptapeta (white) and M. denudata var. purpurascens (violet) flower buds. Peak annotation: 1, Chlorogenic acid; 2, Caffeic acid; 3, Coumaric acid; 4, Rutin; 5, Ferulic acid; 6, Cinnamic acid.

## Slide 3
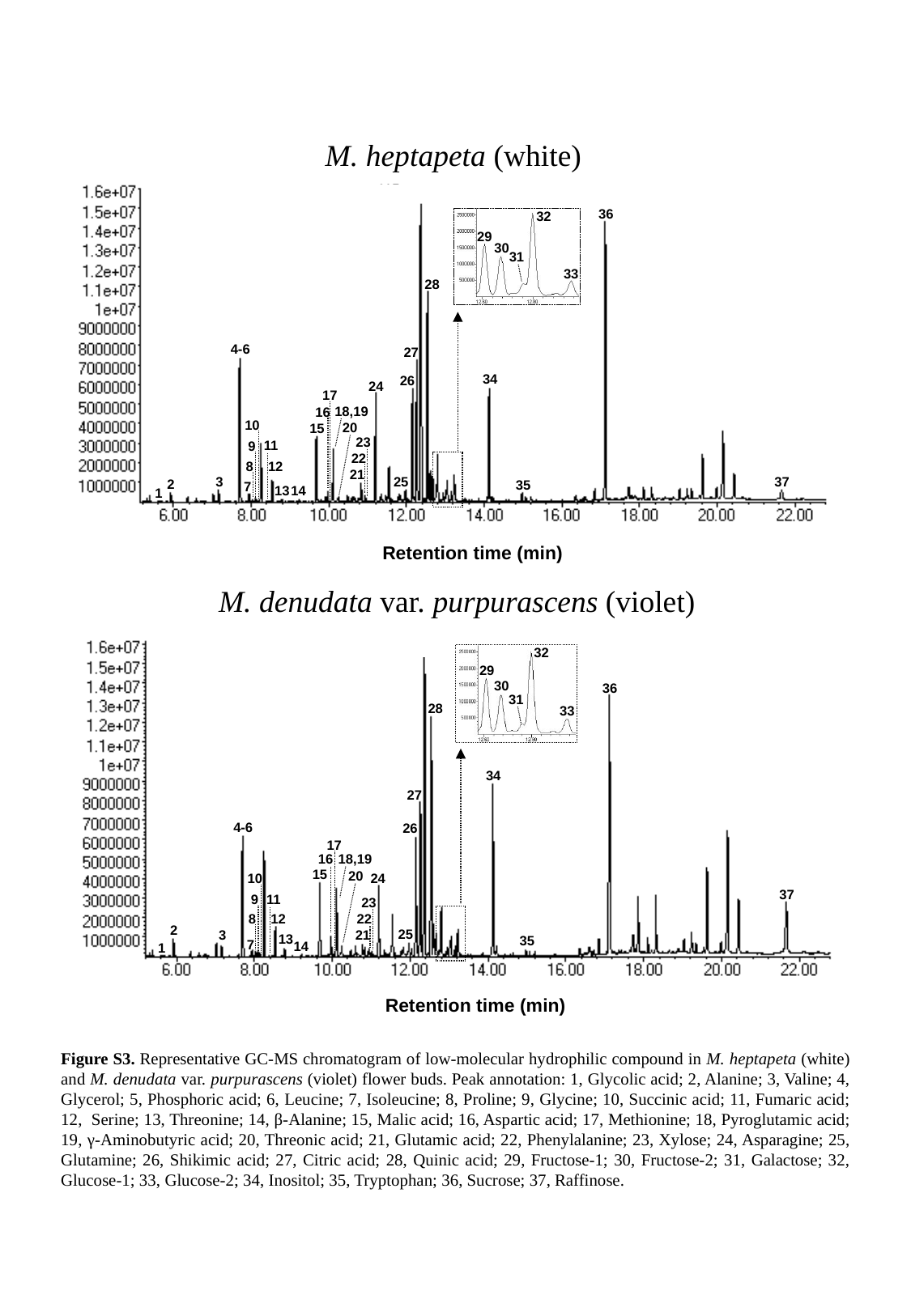

M. heptapeta (white)
36
32
29
30
31
33
28
4-6
27
34
26
24
17
18,19
16
10
20
15
23
11
9
22
8
12
21
3
25
37
2
35
7
14
13
1
Retention time (min)
M. denudata var. purpurascens (violet)
32
29
30
36
31
28
33
34
27
4-6
26
17
18,19
16
15
20
24
10
37
9
11
23
8
22
12
2
25
3
21
13
35
7
14
1
Retention time (min)
Figure S3. Representative GC-MS chromatogram of low-molecular hydrophilic compound in M. heptapeta (white) and M. denudata var. purpurascens (violet) flower buds. Peak annotation: 1, Glycolic acid; 2, Alanine; 3, Valine; 4, Glycerol; 5, Phosphoric acid; 6, Leucine; 7, Isoleucine; 8, Proline; 9, Glycine; 10, Succinic acid; 11, Fumaric acid; 12, Serine; 13, Threonine; 14, β-Alanine; 15, Malic acid; 16, Aspartic acid; 17, Methionine; 18, Pyroglutamic acid; 19, γ-Aminobutyric acid; 20, Threonic acid; 21, Glutamic acid; 22, Phenylalanine; 23, Xylose; 24, Asparagine; 25, Glutamine; 26, Shikimic acid; 27, Citric acid; 28, Quinic acid; 29, Fructose-1; 30, Fructose-2; 31, Galactose; 32, Glucose-1; 33, Glucose-2; 34, Inositol; 35, Tryptophan; 36, Sucrose; 37, Raffinose.

## Slide 4
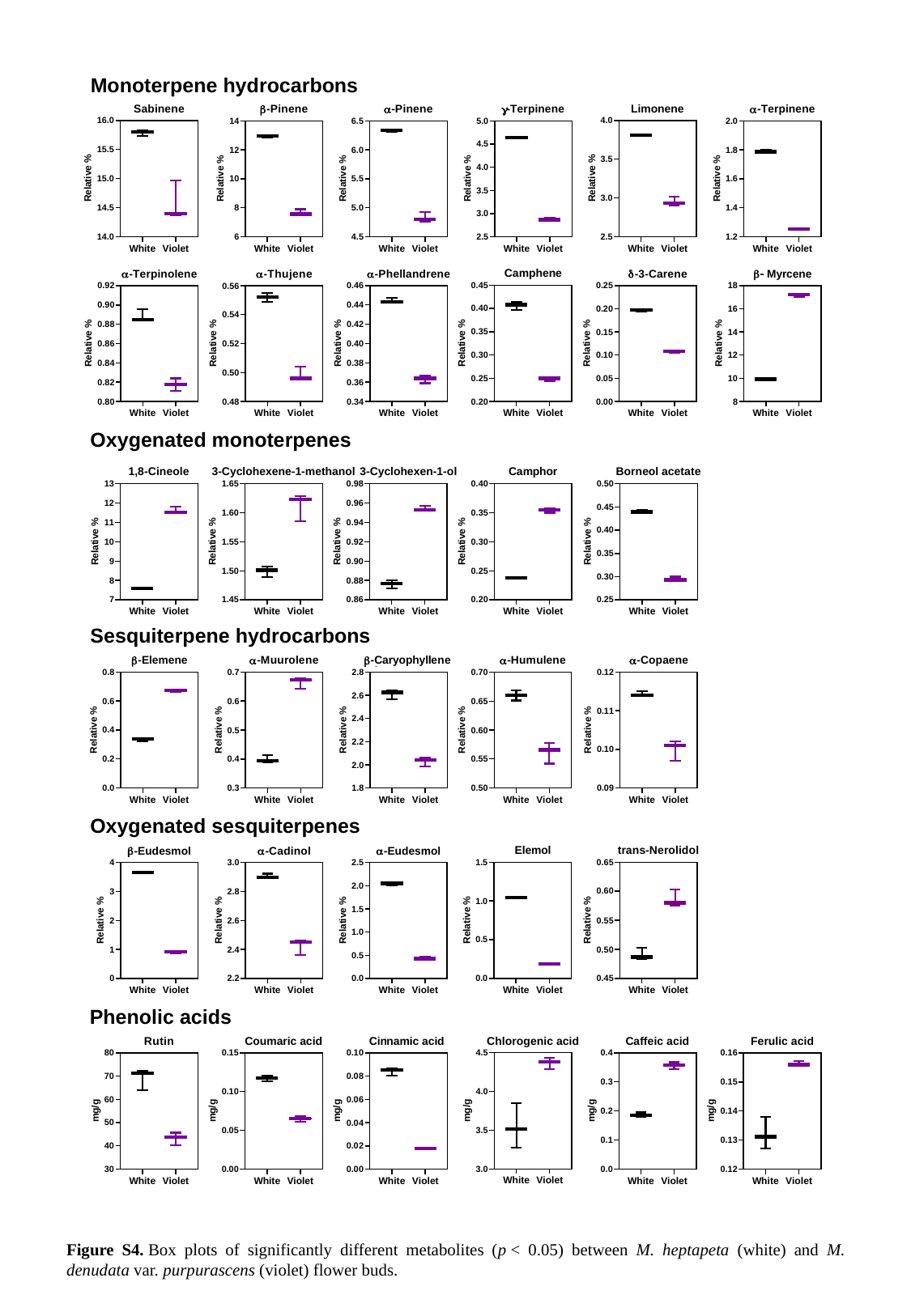

Monoterpene hydrocarbons
Oxygenated monoterpenes
Sesquiterpene hydrocarbons
Oxygenated sesquiterpenes
Phenolic acids
Figure S4. Box plots of significantly different metabolites (p < 0.05) between M. heptapeta (white) and M. denudata var. purpurascens (violet) flower buds.

## Slide 5
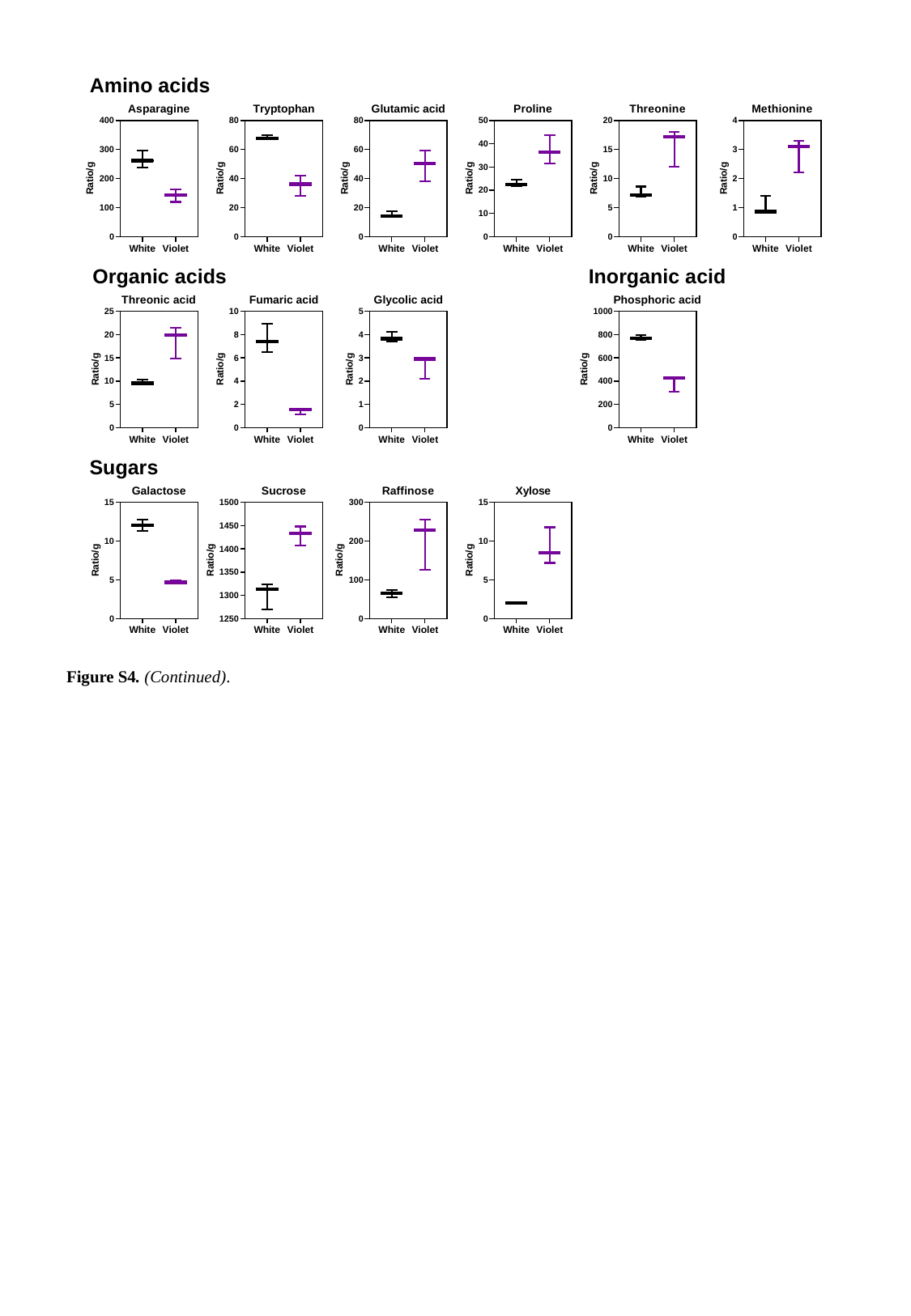

Amino acids
Organic acids
Inorganic acid
Sugars
Figure S4. (Continued).
